# Epidemic Puerperal Fever

**Published:** 1876-01

**Authors:** 


### Epidemic Puerperal Fever.

In the course of some remarks on the above topic Dr. Fordyce Barker (*Medical Record*, Oct. 30, 1875,) said: "I can not resist the conviction that the study of the genesis of puerperal fever is the study of a distinct essential disease which attacks puerperal women, and only puerperal women.

"The great practical end in view in the study of the genesis of puerperal fever is, to ascertain what causes of the disease are preventable. A very great advance has been made within a few years in our knowledge of the various agencies which contribute to septic poisoning, and still more striking has been the addition to our resources in the use of antiseptic remedies. Every intelligent obstetrican appreciates, at the present day, as they did not in former periods, the great importance of averting all the predisposing causes of the disease in the patient herself, by an efficient treatment of anæmia and albuminuria in the last periods of pregnancy, a condition which so tends to blood deterioration, and which so favours the absorption of septic poison—by securing to the patient perfect ventilation and good air during the labour and the puerperal period, avoiding the old error of keeping the room too hot, with ever crevice closed that will admit air; by preventing delay in labour, in the early resort to the use of the forceps or other resources of our art, when necessary; by effecting the early removal of the placenta by compressing the uterus, thus securing the efficient and permanent contraction of this organ, and thus preventing the retention and decomposition of clots, and the torture and exhaustion of after-pains; by removing immediately after labour all soiled clothes and bedding, and carefully watching that none are ever after permitted to contaminate the patient; by antiseptic washes and injections, to prevent autogenetic poisoning; by good nutrition; and lastly, by guarding the patient against the dangers of infection or contagion through the medium of the nurse or the obstetrician. This is a very rapid and by no means complete exposition of the resources we have at command for averting danger from puerperal fever.

"But there still remain, as great determining causes of this fearful disease, nosocomial malaria and epidemy. How to overcome and to exterminate nosocomial malaria is the great problem which I confidently hope will be solved by the progress of science at no remote period. The devastation which results from this cause, in obstetrical and surgical hospitals, has led some to the extreme folly of questioning the usefulness of hospitals, and others to urge as a radical necessity the extravagantly expensive procedure of pulling down all the old hospitals, and of reconstructing them of such material that this process can be repeated ever few years. But, in the first place, this is not demonstrated to be a radical preventative of the septic diseases which result from nosocomial malaria, for there are well-authenticated reports of puerperal fever in new hospitals for maternity, and of pyæmia and of septicæmia in new surgical hospitals among the first patients received into them. In the second place, I cannot believe that chemical science is so powerless as to fail in finding some means of wholly exterminating this miasm. The experiment has been alrealy sucessfully tried in this city. I was struck by the remark in the paper of Dr. Lusk, that after the lying-in wards at Bellevue were given up on account of puerperal fever, they were occupied as surgical wards in the service of Dr. James R. Wood, and that not a single case of septic disease has occurred in them. I am informed by Dr. Dennis, house-surgeon at the present time, that there have been eighteen amputations in patients in these wards, and not a single death. But in some of the surgical wards the fatality from septic disease was really frightful, as reported by the surgeons in attendance; and Prof. Doremus was employed by the Commissioners to disinfect them. I will give his method of procedure, as I wrote it down from his verbal statement to me.

"The purification of the surgical wards in Bellevue Hospital was accomplished during the spring and summer of 1875, by the employment of large volumes of *chlorine* gas.

"This powerful disinfectant was resorted to because all the poisonous emanations from the human system are decomposed by it, and thus rendered inert (carbonic acid gas excepted); also because of its diffusive power. Strips of paper were pasted over the crevices around the windows and doors, before generating the chlorine.

"Two sheets of lead about eight feet long and four feet wide were turned up at their edges and placed on the floor of the ward to be treated.

"In these leaden receptacles several hundred pounds of black oxide of manganese and common salt were placed, to which water was added until the mass, when thoroughly stirred with wooden shovels, had the consistency of a thick mud.

"Bowls, basins, pitchers of sulphuric acid were placed around the leaden vessels in readiness to be applied to the black mixture. To eliminate all the chlorine, the acid should equal the weight of the salt and manganese combined. Water was then poured over the floor to dampen the wood, and the ward was filled with steam until the moisture condensed on the ceiling and walls. The air of the room was so saturated with partly condensed vapour that we had to grope our way towards the vessels containing the sulphuric acid.

"The several assistants then held said vessels over the mixture of manganese and salt, and at a signal all poured out the acid at the same time; then hastened to the second leaden trough, applied the acid and rushed out of the door to escape inhaling the chlorine gas which was liberated in immense volumes. Since the amount of poisonous gas was so great that it would have proved fatal to any one entering the appartment, the doors were securely fastened to guard against such an accident.

"After the lapse of twenty-four hours, the vessels were again filled with sulphuric acid and placed around the leaden pans. The mixture was then rapidly stirred, and the second application of acid made as in the first instance.

"For these two treatments about a carboy of sulphuric acid (160 lbs.) was employed.

"After a second twenty-four hours' exposure of the ward to this gas, the windows were thrown open, the residuum of sulphate of manganese and sulphate of soda was removed, with the leaden and other vessels, and the walls and floor scrubbed and dried.

"The chlorine was generated by this method, rather than by the addition of hydrochloric acid and manganese, not only because it is cheaper, but because the heat generated by mixing sulphuric acid and water rarefies the gas and facilitates its dissemination through the room and its passage into the porous walls.

"Chlorine is comparatively inefficient unless moisture is present, hence steam was employed as described.

"After one ward had been thus disinfected and ventilated, the same large leaden vessels were taken to adjoining ward and the process repeated.

"Especial stress is laid on the importance of generating enormous volumes of the chlorine gas, that it may thoroughly permeate the walls. As its odour is very pronounced, persons are liable to err in regard to the quantity, and they merely produce a bad smell and signally fail to destroy the virus with which old or even new walls are at times impregnated.

"The *water-closets* were perified by the use of *ozone.*

"This active form of oxygen was generated by mixing equal weights of maganate of soda and sulphate of magnesia in a dry state, and sprinkling this mixture in and around the basins at night, so that it might remain for a longer period than if applied in the daytime.

"When brought in contact with water, *permanganate* of soda is produced, which decomposes in contact with the impurities of the sink, and envolves ozone, by which agent the disgusting and poisonous substances are decomposed, deodorized and rendered harmless.

"This treatment was repeated to secure purification.

"One hundred pounds of manganate of soda, and the same weight of sulphate of magnesia, were employed. For generating the chlorine in the different wards over five thousand pounds of the black oxide of manganese, twenty-five sacks of salt and the equivalent of sulphuric acid were used.

"Since this disinfection of the hospital, I am informed by members of the House Staff that there has been but one case of pyæmia or other septic disease in the hospital, and this was a very doubtful one. By the methods adopted by Professor Doremus, or some other method improved by the progress of chemical science, who can doubt that in the future we shall find hospitals, as securely freed from nosocomial malaria as we are now protected from small-pox by vaccination."—*Monthly Abstract.*

